# xenoGI: reconstructing the history of genomic island insertions in clades of closely related bacteria

**DOI:** 10.1186/s12859-018-2038-0

**Published:** 2018-02-05

**Authors:** Eliot C. Bush, Anne E. Clark, Carissa A. DeRanek, Alexander Eng, Juliet Forman, Kevin Heath, Alexander B. Lee, Daniel M. Stoebel, Zunyan Wang, Matthew Wilber, Helen Wu

**Affiliations:** 1 Department of Biology, Harvey Mudd College, 301 Platt Blvd., Claremont, 91711 CA USA; 2 Current address: Department of Genome Sciences, University of Washington, 3720 15th Ave NE, Seattle, 98195-5065 WA USA; 3 Current address: Department of Biology and Biotechnology, Worcester Polytechnic Institute, 100 Institute Rd., Worcester, 01609 MA USA; 4 Current address: Quantitative Biosciences Program, Georgia Institute of Technology, 837 State Street, Atlanta, 30332-0430 GA USA

**Keywords:** Genomic island, Horizontal transfer, Synteny, Gene family

## Abstract

**Background:**

Genomic islands play an important role in microbial genome evolution, providing a mechanism for strains to adapt to new ecological conditions. A variety of computational methods, both genome-composition based and comparative, have been developed to identify them. Some of these methods are explicitly designed to work in single strains, while others make use of multiple strains. In general, existing methods do not identify islands in the context of the phylogeny in which they evolved. Even multiple strain approaches are best suited to identifying genomic islands that are present in one strain but absent in others. They do not automatically recognize islands which are shared between some strains in the clade or determine the branch on which these islands inserted within the phylogenetic tree.

**Results:**

We have developed a software package, xenoGI, that identifies genomic islands and maps their origin within a clade of closely related bacteria, determining which branch they inserted on. It takes as input a set of sequenced genomes and a tree specifying their phylogenetic relationships. Making heavy use of synteny information, the package builds gene families in a species-tree-aware way, and then attempts to combine into islands those families whose members are adjacent and whose most recent common ancestor is shared. The package provides a variety of text-based analysis functions, as well as the ability to export genomic islands into formats suitable for viewing in a genome browser. We demonstrate the capabilities of the package with several examples from enteric bacteria, including an examination of the evolution of the acid fitness island in the genus *Escherichia*. In addition we use output from simulations and a set of known genomic islands from the literature to show that xenoGI can accurately identify genomic islands and place them on a phylogenetic tree.

**Conclusions:**

xenoGI is an effective tool for studying the history of genomic island insertions in a clade of microbes. It identifies genomic islands, and determines which branch they inserted on within the phylogenetic tree for the clade. Such information is valuable because it helps us understand the adaptive path that has produced living species.

**Electronic supplementary material:**

The online version of this article (10.1186/s12859-018-2038-0) contains supplementary material, which is available to authorized users.

## Background

Genomic islands (GI) are clusters of genes that have entered a genome via horizontal gene transfer, that is, outside the normal process of parent-offspring inheritance. Early observations were made in the context of bacterial pathogenicity, where it was found that the difference between pathogenic and non-pathogenic strains often depended on the presence of one or more genomic islands [1]. It soon became clear however, that the function of genomic islands is not restricted to pathogenicity, and that they play a broad role in microbial genome evolution [2–4].

Because of their importance, a significant number of computational methods have been developed for finding GIs. These are distinct from, but fit into a larger literature on finding individual horizontally transferred genes. GI-finding methods can be broadly divided into those that operate on a genome from a single species, and comparative genomics methods that operate on genomes from several species [5, 6].

Many single genome methods are compositional, making use of various attributes of sequence composition such as GC content, oligonucleotide frequency or codon bias. Because genomes differ in these compositional characteristics, when a foreign piece of DNA arrives into a genome, it may differ in some of these characteristics from the genome it is entering. For insertion events that are sufficiently recent, this can be a mechanism to identify foreign DNA. Such methods have been developed to try to take advantage of many compositional features, including GC content [7], oligonucleotide frequencies [8–16], and codon bias [17, 18]. Single genome methods also sometimes target specific sequence features that are associated with GI insertion such as tRNA genes [19]. And a number of such methods use combinations of multiple attributes including composition and/or specific sequence features [20–28].

The basic idea of comparative genomics methods is to compare related genomes to identify regions that are unique to certain genomes and likely result from horizontal transfer. These methods are closely related to methods for identifying the core and pan genomes of a set of species or reconstructing ancestral gene order [29–34]. Comparative methods typically involve whole genome or protein alignments and then some methods built on top of this to identify orthologs and recognize events such as horizontal transfer, deletion, and so on.

Several automated comparative genomics methods for finding GIs have been developed to date. The tRNAcc package combines comparative genomics with a feature specific search [35]. It identifies islands that have inserted near tRNA genes by creating alignments between closely related species using MAUVE [36], and then looking for regions of DNA that are unique to one species near tRNA genes. This approach is good at finding those GIs that insert near tRNA genes, but will miss others. It is included in the web-based MobilomeFINDER service [23].

Another widely used method is IslandPick [37] which has been incorporated into the web service IslandViewer [38–40]. IslandPick is provided with a single input genome where the user desires to find genomic islands. It first identifies a set of comparison genomes, then creates pairwise whole genome alignments using MAUVE, and finally analyzes the alignments to identify regions that are unique in the input genome. This comparative approach allows accurate identification of GIs that are unique to the input genome, and is widely used as a part of the IslandViewer website.

Given continuing reductions in the cost of sequencing, in the future comparative genomics methods are likely to be increasingly important for finding GIs. However existing methods have one important limitation: they are not able to automatically place GIs in the context of a phylogenetic tree. Existing methods such as tRNAcc and IslandPick make use of multiple genomes, but they are best at identifying regions which are unique to one genome compared with others. If we want to study the history of genomic island insertions in a clade of microbes, these methods allow us to find islands that are unique to various strains of the clade. But they do not automatically identify genomic islands which are shared between some strains in the clade, and they do not determine the branch on which those islands inserted within the phylogenetic tree.

Here we describe xenoGI, a system that identifies genomic islands, and maps their origin within a clade of closely related bacteria. Every gene present in a clade has one of two possible origins. Either it originated in the most recent common ancestor of the clade, or it originated in a subsequent horizontal transfer event. The goal of xenoGI is to group genes by origin, identifying islands of genes that entered via horizontal transfer events, and mapping those events onto the phylogenetic tree. Such information is often of interest because it helps us understand the adaptive path that has produced living species.

## Implementation

xenoGI is a command line program implemented in Python. It can be downloaded from http://www.cs.hmc.edu/xgiWeb/ or via GitHub (https://github.com/ecbush/xenoGI).

### Input, output, basic structure

**Fig. 1:**
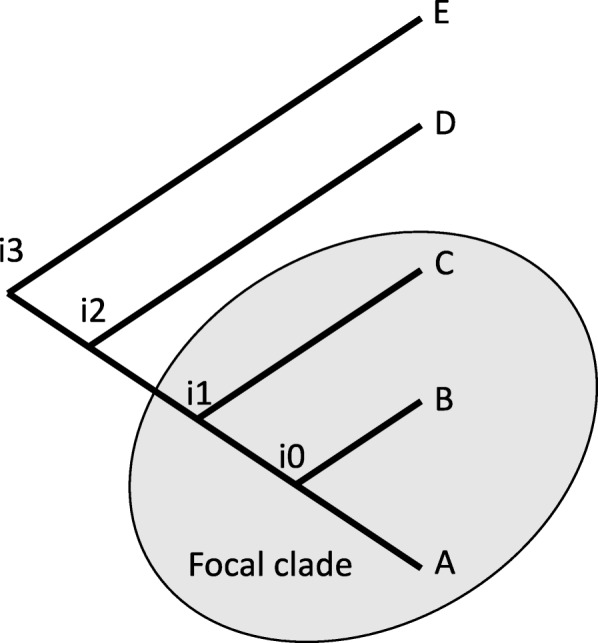
Example species tree. An input tree consisting of a focal clade and several outgroups

Input consists of a set of sequenced genomes in GenBank format, and a tree specifying their phylogenetic relationships. The GenBank files provide protein sequences and their genomic order in each strain. Because the algorithm makes use of synteny information, the genomes need to come from a clade of bacteria that are closely related enough to preserve gene order. For the same reason, the genome assemblies should be at the scaffold level or better. The algorithm is not suitable for analyzing plasmid sequences because of the rapid rate of change of gene content and order on plasmids. Typically, the set of input genomes would include a focal clade that we wish to study, and one or two outgroups (Fig. 1). These outgroups help us to better recognize core genes given the possibility of deletion in some lineages. For cases where the phylogenetic tree is unknown, xenoGI includes optional tools to help users determine it. A provided script allows users to obtain multiple alignments from the set of conserved core genes (discussed below). These alignments can then be used with existing methods to reconstruct a phylogenetic tree.

Every gene in the input genomes must have one of two origins. Either it is a core gene present in the most recent common ancestor of the strains, or it arrived via a horizontal transfer event. The goal of the algorithm is to determine this origin for each gene, grouping genes that arrived together in the same horizontal transfer events as islands. The output is a text file specifying these islands. The output can be visualized further with several text-based visualization functions included in the package, and can also be exported for visualization in a genome browser.

There are three basic steps the algorithm takes. It first calculates a set of scores between genes in the input genomes based on their protein sequences. This includes scores based on sequence similarity and scores based on synteny. It next groups genes into families in a tree-aware way. Finally, it groups these families into islands where the families in an island are interpreted to have a common origin (they either arrived in the same horizontal transfer event or are core genes).

An important aspect of our approach is the use of species tree and synteny information directly in the process of making gene families. The logic is that because we ultimately want to know about the history of GI insertions within a specific species tree, it helps to have gene families that reflect homology due to common decent within that tree, ignoring deeper homology. Every gene family thus formed has a most recent common ancestor that falls on some node in the input tree.

### Calculating scores between genes

The first step is to calculate a set of similarity and synteny scores between genes in the input data set. We initially run protein BLAST between every gene and genes in all the other strains. For those pairs of genes above a certain E-value threshold, we calculate a number of other types of scores.

A raw score is a similarity score between a pair of proteins. We take the global alignment score and scale it to be between 0 and 1, using the following formula:

$$\mathrm{raw\ score} = \frac{g - a }{b-a} $$

where *g* is the global alignment score between two proteins. *a* is a floor value for the alignment score between these two proteins based on what we would get if they were aligned with all gaps (among the pairs we look at, which have significant BLAST hits, there will be nothing lower than this). *b* is a ceiling value for the alignment score (the score of the shorter sequence aligned against itself).

The global alignment is calculated using Parasail [41]. The use of global alignment here reflects the fact that we are operating in a clade of closely related strains and the gene families we build consist of closely related genes. Because of this, we expect alignments between homologs within families to span entire proteins making global alignment preferable to local.

The calculation of raw scores can be run in parallel on multiple processors.

We also calculate a normalized similarity score, which normalizes for the average level of protein distance between a pair of species. Such scores make it easier to set thresholds based on similarity in family formation.

To begin, we identify sets of orthologs where there is exactly one copy in each strain. We do this with the all around best reciprocal hit method, identifying sets of orthologs where every gene is a best reciprocal hit with every other gene, and has one copy in each strain. These sets of orthologs are very conservative and high confidence. We’ll refer to them below as conservative core genes.

Then for each pair of strains we calculate the mean and standard deviation of raw scores between all pairs of orthologs in these sets of conservative core genes. Using this, we take a raw score comparing proteins in two strains and normalize it as follows:

$$\mathrm{norm\ score} = \frac{\mathrm{raw\ score} - m }{s} $$

where *m* is the mean and *s* is the standard deviation of raw scores from the conservative core genes for that pair of strains.

We calculate two kinds of synteny scores based on similarity in the neighborhood of genes.

Our core synteny score makes use of the conservative core genes just discussed. To calculate this score, we define a neighborhood for each gene consisting of a user specified window of conservative core genes in either direction. For example, the neighborhood might be a region encompassing 20 conservative core genes, the first 10 in either direction. To compare a gene A from one strain with a gene B from another, we determine how many conservative core genes from the neighborhood of A are also found in the neighborhood of B, and divide this by the number of conservative core genes in the neighborhood (twenty in the example above). This core-gene synteny score is then:

$$\begin{aligned} &\mathrm{core\ synteny\ score} =\\ &\qquad\qquad\qquad \frac{\mathrm{number\ of\ shared\ genes}}{\mathrm{number\ of\ core\ genes\ in\ neighborhood}} \end{aligned} $$

and is thus a value between 0 and 1.

Because the conservative core gene neighborhoods tend to be large, this measure of synteny looks at a comparatively large region around a gene.

To calculate synteny in a more local region, we also calculate a synteny score based on all genes, including non-core genes.

To compare a gene A from one strain with a gene B from another, we obtain lists of neighboring genes for each. Let *N*_*a*_ be the set of neighboring genes for gene A, taken from within a window of size *W* measured in number of genes, and including both core and non-core genes. Let *N*_*b*_ be the same for gene B. Our local synteny score is calculated as follows. We find the pair of genes with the highest norm score between *N*_*a*_ and *N*_*b*_ and keep it. We remove those genes from *N*_*a*_ and *N*_*b*_, find the next highest pair between them and so on. The local synteny score for gene A and gene B is the average of the *T* highest scoring pairs.

$$\mathrm{local\ synteny\ score} \,=\, \frac{\sum\! T\ \mathrm{highest\ norm\ scores\ from}\ N_{a},N_{b}}{T} $$

The calculation of synteny scores can be run in parallel on multiple processors.

### Forming gene families in a tree-aware way

We wish to build gene families that fit into the known species tree.

For our purposes, a gene family is a set of genes that have descended from a single ancestor gene that existed within that species tree. The most recent common ancestor (MRCA) in such a gene family will fall on the species tree, and its location there will reflect the origin of the family. Gene families whose MRCA falls outside the focal clade are core genes relative to that clade. Gene families whose MRCA falls within the focal clade have arrived via horizontal transfer. This implies that genes that share a deeper homology predating the root of the species tree, will be placed in different families. In cases where the same gene has entered our clade multiple times as a part of distinct transfer events, we want each insertion to correspond to a different gene family.

Our approach is to use a version of the PHiGs algorithm [42] which we have modified to consider synteny information. The PhIGs algorithm operates on the species tree beginning at the root node and moving successively to descendant nodes. At each node, we group all genes descending from species on the left branch (call this group 1), and also group all genes descending from species on the right branch (call this group 2). For nodes other than the root, there are also genes in outgroup species, which we ignore. For every gene in one group, we find the most similar gene in the other, e.g. for all genes in group 1 we find the closest gene in group 2. A pair identified in this way constitutes a seed upon which we build a larger family via single linkage clustering. Because order matters for this approach, the seeds are processed in order of similarity, so that the most similar seeds are worked on first. To build a family from a seed, we identify all previously unclustered genes in either group 1 or group 2 that are closer to a member of the growing family then the original seeds in 1 and 2 were to each other. In our implementation, similarity is measured by the raw score.

We have modified the original algorithm in several ways. In order to add a gene to a family, the basic algorithm requires that it be more similar to some family member than the similarity level of the seed. We have added an absolute threshold for similarity measured using the norm score. This is typically set low, and is a sanity check to make sure we’re not clustering very distantly related proteins together into a family. We also incorporate synteny in a number of ways. We have synteny thresholds for both core and local synteny. Below these thresholds, we do not add a gene to a family. We also use high synteny on the other end, to increase the raw score between a pair of genes we’re considering, potentially helping them get over the bar of seed similarity set by the basic algorithm.

This step runs on a single processor.

### Grouping families into islands

Our goal is to group gene families that arrived together as a part of the same horizontal transfer event. We refer to such groups as islands.

We first sort families according to their MRCA because families that belong in the same island will have the same MRCA. We then build islands using a greedy approach that progressively adds families that are inferred to be adjacent to each other in the MRCA.

We identify genes to add to an island using a parsimony-based metric. Our metric uses a very simple notion of evolutionary changes in gene order. If a pair of genes were adjacent, but due to rearrangements move apart, we assess a cost of one. Similarly if two genes were non-adjacent, but due to rearrangements move to be adjacent, we also assess a cost of one. Consider two gene families with the same MRCA, for example i0 in Fig. 1. We have adjacency information for those families in species A and species B. Our approach is to calculate a rearrangement cost for these families under two cases: either assuming they were adjacent at their MRCA, or assuming they were not. We define the rearrangement score for the families as follows:

$$\begin{aligned} &\mathrm{rearrangement\ score} =\\ &\qquad\quad\mathrm{cost\ starting\ non-adjacent} - \mathrm{cost\ starting\ adjacent} \end{aligned} $$

This score can range between -2 and 2 with more positive values indicating families that are more likely to have been adjacent in the common ancestor.

We begin by creating a set of one-family islands for all families with a particular MRCA. We then calculate the rearrangement score for every pair of islands and identify the pair with the highest score, arbitrarily breaking ties. Then we merge this pair into a two-family island and recalculate its rearrangement score with other islands. Note that because multi-family islands have a gene order (the inferred order in the MRCA), to calculate rearrangement scores for them, we consider adjacency on each of their two ends. The algorithm continues merging islands until all the rearrangement scores are below a certain threshold. It then repeats the procedure relaxing the criterion for adjacency (e.g. we can count as adjacent genes that are one gene away from each other). When all the rearrangement scores are below threshold, the algorithm terminates.

The island-making step runs on multiple processors.

### Analysis tools

Included in the package are command line tools for identifying and visualizing islands at particular nodes, for finding islands associated with particular genes and for examining gene families. Also included are scripts for exporting xenoGI island output to bed or gff format for visualization in a genome browser such as IGB [43].

## Results

### Validation via simulation

We assessed the effectiveness of xenoGI in two ways: using simulations, and detecting known genomic islands.

We used simulations to produce test data sets where the location of GI insertions within a phylogenetic tree was known. To do this we implemented a custom simulator that evolved sequences over a user provided phylogenetic tree, allowing for horizontal transfer of novel genes (from outside the clade) as well as for genomic scale deletions, duplications and inversions, and amino acid level sequence change. The latter was done using the pyvolve module [44].

**Fig. 2:**
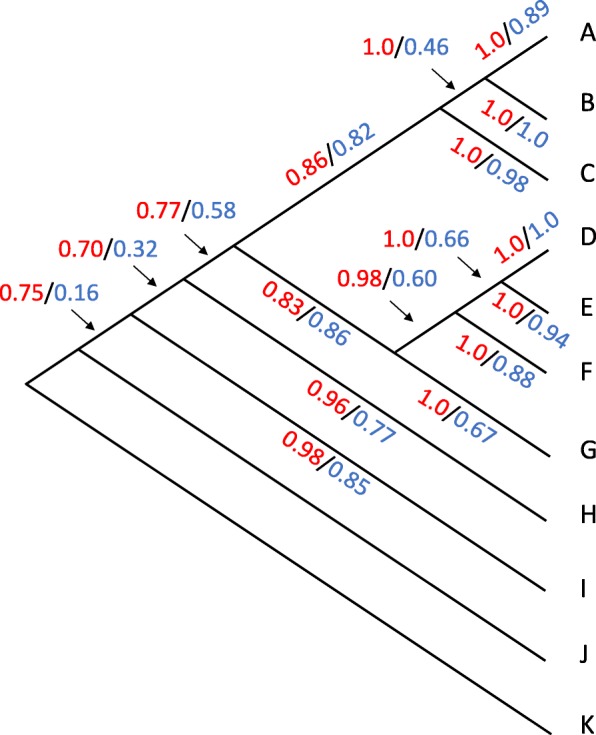
Phylogenetic tree used in genome simulations. We ran xenoGI on simulated genomes that were generated on the tree shown. On each branch we show the true positive rate (red) and the positive predictive value (blue) for xenoGI on that branch

Figure 2 shows the results of a simulation on a tree with 11 species. The simulation was run on a tree matching the branch lengths and topology of a tree from the enteric bacteria, discussed below. Species A-I form the focal clade, with J and K as outgroups. The simulation contained a total of 495 horizontal transfer events which mapped onto the various branches. These ranged in size from 2 to 51 genes. There were also 1009 deletions (from 1 to 50 genes in size), 494 duplications (from 1 to 48 genes in size), and 125 inversions (from 5 to 147 genes in size).

xenoGI achieved a base-wise true positive rate of 0.85 and a positive predictive value of 0.51 over the whole tree. What we mean by base-wise, taking the example of the true positive rate, is that among all nucleotides that were truly in genomic islands, xenoGI correctly identified (and placed on the correct branch) 0.85 of them. The majority of islands (0.83) had just a single xenoGI predicted island overlapping them. As can be seen from the figure, xenoGI’s accuracy is best for GIs inserting on tips, and declines as we move to internal branches.

### Validation via GIs from the literature

Simulations have the advantage of providing a situation where ground truth is known. However they are necessarily simplified, and may not adequately capture the features of real genome evolution. For this reason, we also examined how well xenoGI identified genomic islands that have been reported in the literature.

Wei et al. compiled a list of known genomic islands from 11 bacterial strains for the purposes of validation [28]. We used six of these strains for testing as we built the system, and for developing a set of default parameters. We held the five remaining strains as a validation data set, the results of which we report here. For each of the five strains, we identified five or six closely related species with genomes in GenBank. We reconstructed the history of genomic island insertions in the resulting clade using xenoGI with default parameters. A listing of the strains and trees used here can be found in Additional file 1. We note that there were not many cases where these known islands were shared among multiple already sequenced genomes. Thus most of the cases we look at here, the islands are on the tips of the phylogenies we created. In the section below we give several examples of cases where islands can be identified on internal branches of a tree.

The islands in this validation set were reported in genome papers for their respective strains [45–49]. They are chiefly based on comparative work involving human curation, and in some cases on experimental evidence as well. They are likely to represent true islands. However we cannot be sure that these represent exhaustive lists of all genomic islands in a strain. For this reason, it does not make sense to calculate a true positive rate or positive predictive value here. The validation ranges are given in nucleotide positions, whereas xenoGI identifies genes that are part of an island. For the purposes of comparison, we consider the nucleotides of a xenoGI island to be those between the beginning of the first gene and the end of the last gene.

Table 1 shows our results for this analysis. As can be seen from the table, the base coverage, that is the proportion of validation island bases that are covered by a xenoGI island, is in the upper 90 percent range for 4/5 strains, and 88% for Cronobacter. In addition, in the majority of cases, xenoGI identified a single island corresponding to each validation island. And in most cases (6/11) where a validation island was broken into more than one xenoGI island, this was actually correct, due to the fact that the validation island had genes with a most recent common ancestor at different times on the tree, and likely resulted from multiple horizontal transfer events.

**Table 1:** Summary of xenoGI results on validation cases in five strains

	Num. islands	Total bases	Base coverage	In single island
Burkholderia cenocepacia J2315	13	530,772	0.977	0.917
Corynebacterium diphtheriae NCTC 13129	13	249,918	0.983	0.769
Cronobacter sakazakii ATCC BAA-894	14	305,124	0.879	0.643
Streptococcus equi 4047	7	243,337	0.951	0.857
Vibrio cholerae O1 biovar eltor str. N16961	6	295,096	0.970	0.833

Each row corresponds to a strain. Num. islands represents the number of validation islands and total bases represents the total number of nucleotides in those islands. Base coverage is the proportion of all bases in the validation islands that xenoGI correctly recognized as an island. In single island indicates the proportion of validation islands that xenoGI captured as a single island

Additional file 1 contains a more detailed description of our comparison between xenoGI islands and validation islands. One of the measures included is extra coverage, which refers to cases where a xenoGI island extends beyond the range of the validation island. There were 21 cases where the extra coverage was more than 10% of the length of the island. In the majority of these (16/21) further examination suggested that in fact xenoGI was correct to extend the range of the island. Additional file 2 contains comparison results with several widely used tools from the IslandViewer web site [18, 19, 22, 37, 40]. These tools have been run on the same five species used in Table 1, and using the same two metrics. The file shows that xenoGI’s performance compares favorably with these tools. For both base-wise coverage and the proportion validation ranges covered by a single predicted island, xenoGI has values greater than (or in one case equal to) those for the other methods.

### Reconstructing the timing of GI insertions: two examples from enteric bacteria

**Fig. 3:**
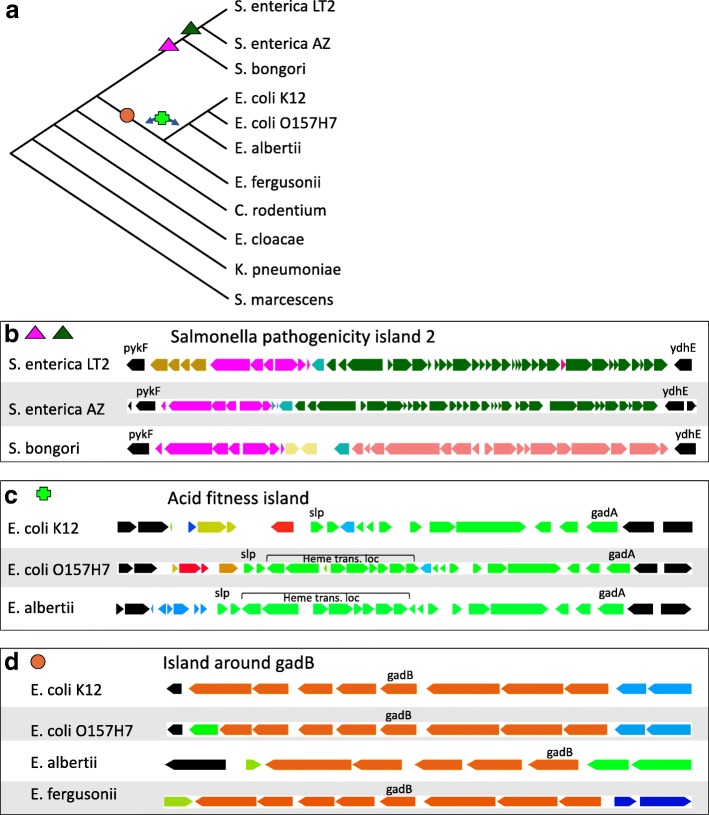
Examples from enteric bacteria. **a** Phylogenetic tree of 11 enteric species. Symbols indicate the branches of insertion of GIs in **b**–**d**. The images in **b**–**d** were made by outputting xenoGI islands and then displaying in the IGB genome browser. Note that the scale for the three is not exactly the same. In the figures, different islands are given different colors. All islands with an mrca at or before the point where *C. rodentium* diverges are colored black. **b** Salmonella pathogenicity island 2 shown in three *Salmonella* species. **c** The acid fitness island as reconstructed by xenoGI in two *E. coli* species and *E. albertii*. **d** The island around *gadB* in our four *Escherichia* species

We look at two examples of genomic islands from the enteric bacteria with the goal of demonstrating the sort of analysis one can do with xenoGI. We do this on a tree of eleven enteric species (Fig. 3a).

The first example is Salmonella pathogenicity island 2 (SPI-2), from *Salmonella enterica* [50, 51]. This island is essential for virulence in *S. enterica* and contains a type-III secretion system that is expressed while the bacterial cell is inside host cells [52]. The island was originally defined as the region between but not including the genes *ydhE* and *pykF* [53]. It is known to consist of several components with different evolutionary origins [54].

xenoGI’s results are consistent with what we would expect from the literature. The largest part of SPI-2 is colored dark green in Fig. 3b. It includes the type III secretion system and is shared between *Salmonella enterica* strains (LT2 and Arizoniae in our sample), but is not present in *Salmonella bongori*. xenoGI puts this region in one island, reflecting the common origin of the genes. All the genes in the region are included, with the exception of one gene specific to *S. enterica* LT2 which is shown in red. xenoGI identifies another island (shown in pink) corresponding to a second part of SPI-2. This region contains the tetrathionate reductase gene cluster. Consistent with the literature, xenoGI identifies this region as being shared between *S. enterica* and *S. bongori* [54]. The final part of SPI-2, shown in brown in Fig. 3b, contains genes that are present in *S. enterica LT2*, but not *S. enterica arizoniae*.

Our second example is the acid fitness island (AFI) in *E. coli* [55]. This island runs from slp to gadA inclusive in *E. coli* K-12 (Fig. 3c) and is about 15 kb long [56]. It encodes a number of genes involved in resistance to acid stress including a glutamate decarboxylase enzyme (coded for by *gadA*) and its regulators. xenoGI identified the island from *slp* and *gadA*, and placed it on the branch after the divergence of *E. fergusonii*, but before the divergence of *E. albertii*. One internal gene which is part of the island, *yhiD*, was left out because it is not present in *E. albertii*.

Further exploration reveals some additional features of the evolution of acid fitness genes in *Escherichia*. The acid fitness island was originally described in *E. coli* K-12 [55], and contains 12 genes in that strain. However the island in both *E. coli* O157:H7 and *E. albertii* contains an additional 8 genes. As it turns out, these additional genes, which are involved in the scavenging of iron from hosts, have been identified and studied previously. They were identified first in *Shigella dysenteriae*, but were found to also be present in a number of *E. coli* strains [57]. (It’s worth noting that *Shigella* strains nest within the *E. coli* clade.) This so-called heme transport locus falls in the middle of the AFI, but because the AFI has mostly been studied in *E. coli* K-12, and that strain lacks the heme transport locus, it was never recognized that the two are co-localized. xenoGI places them in the same island because they have the same most recent common ancestor, being present in some *E. coli* strains and *E. albertii*. It is possible that these two sets of genes with seemingly distinct functions arrived as a part of a single event.

A second question relates to the time of arrival of the AFI. xenoGI places it on the branch before *E. albertii* diverged (Fig. 3a). However, an analysis of AFI homologs in other strains using several functions included in the package reveals that *E. fergusonii* contains a nearly complete copy of the AFI (missing only the heme transport genes and *gadA*), but in a non-syntenic location. The question then is whether this island in *E. fergusonii* represents an independent insertion. Alternatively, it may have resulted from the same insertion as in the other *Escherichia* strains, but have been moved to a different location via some rearrangement or translocational process. It has been observed that *E. fergusonii* has undergone a large number of genome rearrangements since the time of its divergence from *E. coli* [29]. That notwithstanding, the complete lack of synteny here favors an independent insertion, and is the reason xenoGI placed *E. fergusonii’s* AFI in a separate island.

Another issue is the phylogenetic distribution of the heme transport genes. The original paper on these genes observed a puzzling phylogenetic distribution in *E. coli* [57]. We observe something similar in our enteric species. The heme transport locus is not present in *E. coli* K-12 and *E. fergusonii*, but is present in *E. coli* O157:H7 and *E. albertii*. This distribution would require an insertion into and then a clean deletion from the AFI, or else some process involving horizontal transfer and perhaps gene conversion.

The status of another set of genes can also shed light on the evolution of acid tolerance in *Escherichia*. *Escherichia* genomes contain a second glutamate decarboxylase enzyme, *gadB*. *gadB* has typically been seen as the result of a gene duplication, the idea being that the AFI was inserted, and then *gadB* arose by duplication [58]. However xenoGI finds that the *gadB* gene lies in an island consisting of 8 genes that is shared by the entire *Escherichia* clade (Fig. 3d), including *E. fergusonii*, but is not found outside that group. That is, it appears to have arisen on the branch leading to *Escherichia* before the divergence of *E. fergusonii*. This fact raises the question as to whether *gadB* is really a duplication or the result of an independent insertion event (albeit one that may have been followed by gene conversion events between *gadA* and *gadB* [58]). The other genes in the island surrounding *gadB* do not have close paralogs in other parts of the genome, a fact which may favor the idea of an independent insertion via horizontal transfer.

Acid tolerance has often been seen as a feature unique to *E. coli* [58], however our results show that this is in fact a characteristic of the whole *Escherichia* clade. This fact does have some practical significance, as AFI genes have been the basis for assays attempting to identify *E. coli* in samples [59, 60]. Beyond this our data suggest that the evolution of acid fitness in this group was more complicated than previously appreciated, likely involving multiple insertion events.

### Resource usage

We have run xenoGI on up to 115 strains. There is a tradeoff between time and RAM usage, because using more processors requires more RAM. Additional file 3 shows plots of RAM usage, user time and wall clock time for up to 40 strains running on 50 processors. On our machine, 40 strains required approximately 500 GB of ram and around 20 h from start to finish.

## Discussion

The results presented above show that xenoGI performs well on both simulated and real data. In the validation using simulations, xenoGI’s true positive rate and positive predictive value were high, and most insertions were recognized as a single island. We did observe that xenoGI’s effectiveness declined as we moved to more internal branches. This trend is not surprising. Internal branches are older, and in the extra time that has passed, events may have occurred that obscure the evidence for a horizontal transfer event. xenoGI also correctly identified most of the previously identified validation islands in real genomes (Table 1). Often in cases where it seemingly made a mistake, e.g. split a single validation range into several islands, upon closer examination we found that its result was correct (Additional file 1). Furthermore, xenoGI performs favorably compared with several widely used previous methods [18, 19, 22, 37, 40]. It has higher values for both base coverage and the proportion of a validation range represented by a single island (Additional file 2). We note that in assessing the effectiveness of xenoGI and other packages, we have chosen not to use metrics such as the true positive rate or the positive predictive value. This is because the validation ranges we are using are unlikely to represent an exhaustive list of all GI’s in those species. Metrics like the true positive rate and the positive predictive value require an exhaustive list. Finally, our examples from enteric bacteria illustrate the novel aspect of xenoGI’s functionality, the ability to identify genomic island insertions that have occurred on internal branches of the tree for a set of strains. Taken as a whole, these results show that xenoGI can effectively reconstruct genomic island insertions in clades of closely related bacteria.

One topic it is worth addressing briefly is the relationship between the number of strains used and the effectiveness of xenoGI. In general, we have found that as we increase the number of strains, the system becomes better at accurately placing genes in families. However, when the number of strains becomes too large (on the order of dozens) it has a tendency to inaccurately break islands on deep internal branches into multiple parts.

As a comparative genomics approach, xenoGI is relatively resource intensive compared with methods that examine composition in a single genome. However, in return it provides a comprehensive assessment of the history of GI insertions into a clade. The fact that it operates in the context of a clade makes it distinctive compared with previous automated comparative genomics methods. Other distinctive features include the fact that it is gene based, doesn’t depend on MAUVE, and integrates species tree and synteny information from an early stage of its analysis. In the past, reconstructing the history of GI insertions into a clade typically required heavy human involvement. Our hope is that xenoGI will make this sort of thorough comparative approach accessible to more users.

It is also worth reflecting briefly on some of the assumptions and limitations of our approach. To create gene families, xenoGI makes extensive use of synteny information. In situations where synteny is poor due to changes in gene order and composition, then family formation will be inaccurate. If the reduction in synteny is moderate, it is possible to adjust the parameters to compensate for this to some extent, e.g. by shrinking the synteny window sizes. However, in sequences such as plasmids which have undergone rapid changes in gene order and composition, the system does not perform well.

Another caveat has to do with genomic island insertion hotspots. It has been observed that certain regions are more likely than others to receive insertions [29]. When there are multiple insertions of the same or very similar GIs, xenoGI distinguishes these insertions using synteny. However, if similar islands insert multiple times in the same region, it will not be able to recognize those events as distinct.

Future work might attempt to use additional information such as that available in gene trees to supplement decisions we are currently making based on synteny. This could potentially enable us to better recognize cases where related islands inserted in the same location multiple times.

More generally, the problem of making tree-aware families is one that might be aided with machine learning approaches. The creation of such families involves integrating multiple pieces of information. However it is challenging to create a single set of rules that captures what we want to do. It may be easier for a human to annotate a clade of bacterial genomes, creating a set of gene families, and then let a machine learning algorithm learn from that. The algorithm would be using similar information to our current algorithm, but would have learned the rules based on a training set.

Finally, genomic islands which have inserted multiple times in different strains seem to be relatively common. The AFI discussed above is a potential example. It would be helpful to have a more systematic way to identify such cases. To do this, we could potentially add an additional step to xenoGI which involves comparing all the islands found in a clade against each other.

## Conclusions

As more and more microbial genomes are sequenced, it becomes desirable to analyze genomic adaptation in the context of phylogenetic trees. Here we have presented xenoGI, a software package that takes a clade of closely related microbes and identifies islands of genes that entered via common horizontal transfer events, placing those events on the phylogenetic tree for the clade.

## Additional files

Additional file 1 A more detailed description of the correspondence between xenoGI islands and validation islands. A tab-delimited text file containing each validation range used in the five species we looked at. Includes the assemblies used to compare with each strain and the tree. For each validation range gives the base-wise coverage, the amount of extra coverage, the number of overlapping xenoGI islands and any comments. (TSV 6 kb)

Additional file 2 Comparisons with commonly used GI finding software. A tab-delimited text file containing comparisons between xenoGI and four other commonly used GI finding programs: SIGI-HMM, IslandPick, IslandPath-DIMOB, and Islander. These were done using validation ranges from the same five species used for in Table 1 and Additional file 1. Included are data for base-wise coverage and the proportion of validation ranges covered by a single predicted island. (TSV 0.834 kb)

Additional file 3 Resource usage of xenoGI. Plots of RAM usage, user time and wall clock time for up to 40 strains running on 50 processors. (PDF 5 kb)

## Abbreviations

AFI: Acid fitness island
GI: Genomic island
MRCA: Most recent common ancestor
