# Supplementary figures and images for: xenoGI: reconstructing the history of genomic island insertions in clades of closely related bacteria
Source: BMC Bioinformatics. 2018 Feb 5;19:32. doi: 10.1186/s12859-018-2038-0 (PMC5799925; doi:10.1186/s12859-018-2038-0)

**User time**

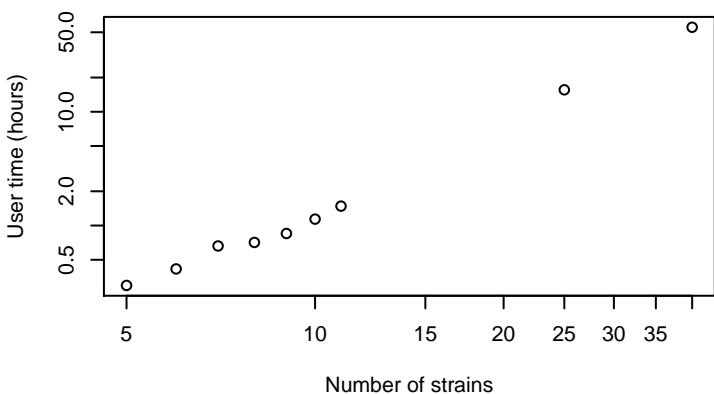

**Wall clock time**

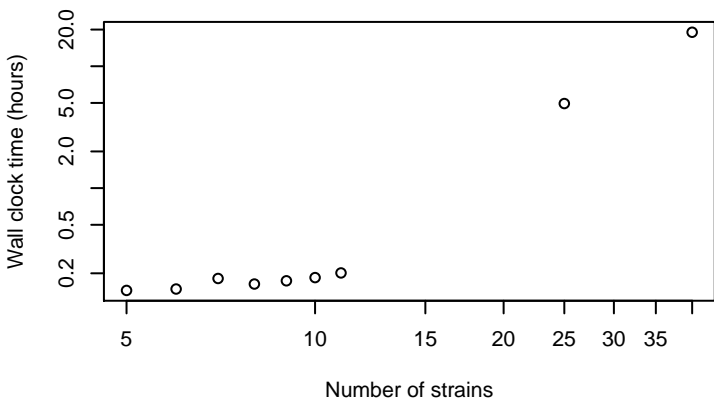

**RAM usage with 50 threads**

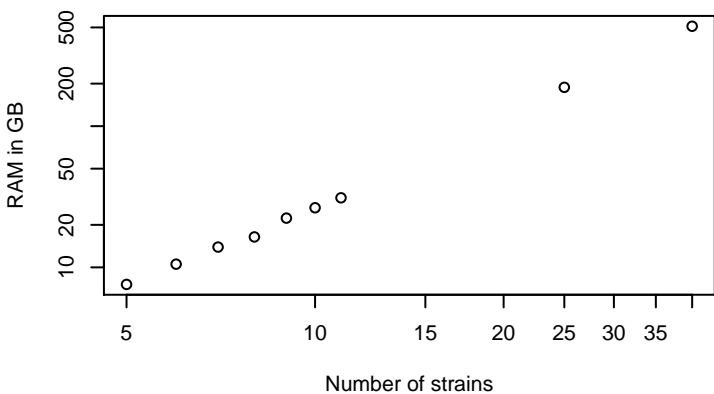

Supplement: Supplementary file 3 — Resource usage of xenoGI. Plots of RAM usage, user time and wall clock time for up to 40 strains running on 50 processors. (PDF 5 kb) [file 12859_2018_2038_MOESM3_ESM.pdf]
